# Mechanisms of oral absorption improvement for insoluble drugs by the combination of phospholipid complex and SNEDDS

**DOI:** 10.1080/10717544.2019.1686086

**Published:** 2019-11-18

**Authors:** Yingpeng Tong, Qin Zhang, Wen Shi, Jianxin Wang

**Affiliations:** a School of Advanced Study, Institute of Natural Medicine and Health Product, Taizhou University, Taizhou, China;; b Department of Pharmaceutics, School of Pharmacy, Ministry of Education, Fudan University & Key Laboratory of Smart Drug Delivery, Shanghai, China;; c Institute of Integrative Medicine, Fudan University, Shanghai, China

**Keywords:** Silybin–PC–SNEDDS, oral bioavailability, absorption mechanism, lymphatic transport

## Abstract

In the present study, a water insoluble drug named silybin was encapsulated into self-nanoemulsifying drug delivery system (SNEDDS) following the preparation of silybin–phospholipid complex (SB–PC), then several methods were carried out to characterize SB–PC–SNEDDS and elucidate its mechanisms to improve the oral absorption of SB. Using a dynamic *in vitro* digestion model, the lipolysis of SB–PC–SNEDDS was proved to be mainly related with the property of its lipid excipients. SB–PC–SNEDDS could significantly enhance the transport of SB across Caco-2 cells, which may partly attribute to the increased cell membrane fluidity and the loss of tight junction according to the analysis results of fluorescence anisotropy of 1,6-diphenyl-1,3,5-hexatriene (DPH) and tight junction protein (ZO-1). The result of *in situ* perfusion showed the intestinal absorption of SB from high to low was SB–PC–SNEDDS, SB–PC, and SB. The extent of lymphatic transport of SB–PC and SB–PC–SNEDDS via the mesenteric duct was 12.2 and 22.7 folds of that of SB, respectively. In the lymph duct cannulated rats, the relative bioavailability (Fr) of SB–PC and SB–PC–SEDDS compared to SB was 1265.9% and 1802.5%, respectively. All the above results provided mechanistic support for oral absorption improvement of water insoluble drugs by the combination of PC and SNEDDS.

## 1. Introduction

Oral administration is considered as the most favorable administration that is often convenient, painless, and noninvasive. Unfortunately, certain drugs and bioactive molecules with low bioavailability are not suitable for this administration because of their poor solubility, low permeability, and chemical instability. In recent decades, tremendous efforts have been paid to tackle the challenges. Among them, phospholipid complex (PC) is a common method to increase bioavailability of free drug, which has been confirmed by animal and clinical pharmacokinetics and activity studies (Mirzaei et al., 2017; Qian et al., 2018; Biswas et al., 2019; Qian et al., 2019). However, due to the high viscosity and poor water-solubility, the low dispersion and dissolution of PC in gastrointestinal (GI) fluid will limit drug’s absorption (Jiang et al., 2016; Yang et al., 2018). In order to overcome these problems, some approaches have been developed to by further preparing PC into oil solution, lipid nanocarriers, nanoparticles, and so on. Compared with above technologies, researchers have focused more on self-nanoemulsifying drug delivery system (SNEDDS) because of its thermodynamic stability, ease of preparation and significant improvement in solubility (Khatri & Shao, 2017). SNEDDS, a mixture of drug, oil, surfactant, and co-surfactant, can generate oil-in-water (O/W) nanoemulsion with the droplet size less than 100 nm when gently mixed with water (Xue et al., 2018; Rani et al., 2019). These fine droplets could enhance the dispersion of drug dissolved inside the oil phase into GI fluid, resulting in the significant improvement of absorption in GI tract (Rehman et al., 2017). In turn, PC can also help SNEDDS to overcome its limitation. For a portion of compounds without sufficient liposolubility, they may fail to be directly entrapped into SNEDDS because of the insufficient lipid solubility which can be improved by PC (Ding et al., 2018). All in all, the combination of PC and SNEDDS is an innovative way to improve the oral absorption of insoluble drug.

Some attempts have been carried out by combination of PC and SNEDDS to enhance oral bioavailability of bioactive compounds or biomacromolecule, such as morin (Zhang et al., 2011, 2015, 2016; Li et al., 2019), akebia saponin D (Shen et al., 2016; Wang et al., 2019), rosuvastatin calcium (Beg et al., 2019), paclitaxel (Ding et al., 2018), curcumin (Shukla et al., 2017), ellagic acid (Avachat & Patel, 2015), baicalin (Wu et al., 2014), matrine (Ruan et al., 2010), and insulin (Zhang et al., 2012). Most of above researches focused on preparation, characterization, bioavailability, or pharmacodynamics of the PC–SNEDDS complex. A few reports studied the mechanisms of bioavailability improvement by PC–SNEDDS complex and attributed these effects to increased membrane permeability (Zhang et al., 2015; Wang et al., 2019), P-gp inhibition (Zhang et al., 2015), destroying self-micelles (Wang et al., 2019), and inhibiting the intestinal metabolism (Wang et al., 2019), but the PC–SNEDDS complex could not bypass first-pass metabolism which will compromise the enhancement of oral absorption (Li et al., 2019). In summary, the underlying exact mechanisms on how PC–SNEDDS complex to improve the absorption of drugs remains unclear and needs to be clarified.

**Figure 1.**
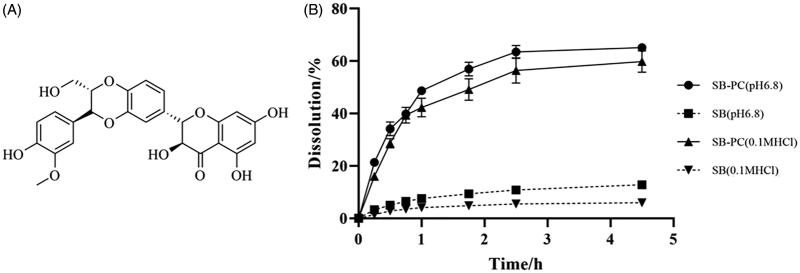
The chemical structure of SB and its cumulative release profiles in 0.1 M HCl and pH 6.8 PBS (*n* = 3).

*Silybum marianum* is a famous herbal medicine widely used in treating liver diseases for 2000 years. Flavonolignan silybin (Figure 1(A)) is considered as its main active ingredient. The action of silybin in the treatment and prevention of liver disorders can be mainly contributed to its antioxidant and anti-inflammatory activity, as demonstrated *in vitro* and *in vivo* (Federico et al., 2015; Ou et al., 2018; Zhang et al., 2018). It receives more and more attentions recently due to the new activities in nontraditional applications, such as anticancer (Zhang et al., 2016; McCormick et al., 2018), neuroprotective activity (Ares et al., 2018), skin protection (Svobodova et al., 2018), etc. However, served as a BCS class II drug, the poor water solubility and intestinal absorption capacity of silybin, resulting in very low bioavailability, will hamper its possible therapeutic applications. Silybin–phospholipid complex (SB–PC) has been developed and could give significantly higher plasma levels by oral administration compared with silymarin or silybin according to the studies on rats (Yanyu et al., 2006; Duan et al., 2011; Angelico et al., 2014) or human beings (Abenavoli et al., 2015; Malaguarnera et al., 2015; Nahum et al., 2019). In addition, SB–PC has been demonstrated to be well tolerated in preclinical and clinical studies (Barzaghi et al., 1990). Therefore, silybin was chosen as a model drug in this study to elucidate the absorption improvement mechanisms of PC and its SNEDDS.

After oral administration, the PC and its SNEDDS will rapidly pass through the oral cavity and esophagus, then enter the gastric cavity, and finally be transported into systemic circulation and various tissues by absorbing through the small intestine. According to mechanisms of oral administration (Khatri & Shao, 2017; Rehman et al., 2017; AboulFotouh et al., 2018; Beg et al., 2019; Wang et al., 2019), the enhanced bioavailability of PC–SNEDDS may attribute to the improvement of drug solubility and permeability, the inhibition of intestine metabolism and P-gp efflux, the changes of GI tract (such as opening the tight junctions) and the stimulation of intestinal lymphatic transport. Therefore, in order to explore the mechanisms of oral absorption improvement for water insoluble drugs by the combination of PC and SNEDDS, the digesting process of the formulations were investigated by *in vitro* dynamic lipolysis model. The permeability and absorption rate of the formulations and their transport mechanisms from epithelial cells were explored by Caco-2 and single-pass intestinal perfusion (SPIP) technique. Furthermore, an unconscious rat model was employed to study the lymphatic transport of the formulations. The above results can provide theoretic support for research and development of natural drug–PC, promote the application of PC and SNEDDS in the improvement of oral bioavailability of water insoluble drugs.

## 2. Materials and methods

### 2.1. Materials

Silybin coarse powder (purity: 98.0%) was provided by Panjin Huacheng Pharmaceutical Co., Ltd. (Panjin, China). The reference substance of silybin (purity >99%) was purchased from National Institute for the Control of Pharmaceutical and Biological Products (Beijing, China). Soybean phospholipid with the phosphatidylcholine (PC) content of approximately 98% was supplied by Shanghai Tai-Wei Pharmaceutical Co. Ltd. (Shanghai, China). Labrasol and transcutol HP were gifts from GATTEFOSSE (Saint-Priest, France). Cremophor RH40 was a gift from BASF (Ludwigshafen, Germany). Dulbecco's modified Eagle medium (DMEM, high glucose), non-essential amino acids, fetal bovine serum, and Hank's buffered salt solution (HBSS) were bought from Gibco Company (Carlsbad, CA). Methanol of HPLC grade was obtained from TEDIA Company, Inc. (Fairfield, CT). Water was purified by using a Milli-Q water purification system (Millipore, Bedford, MA). All other reagents and chemicals used in this study were of analytical grade or better.

### 2.2. HPLC method for the determination of SB

#### 2.2.1. Chromatographic conditions

The SB was determined by HPLC system Agilent 1200 coupled with a reversed-phase Luna C_18_ (5 μm, 250 × 4.6 mm). The mobile phase of the HPLC system consisted of methanol and 0.05 M KH_2_PO_4_ aqueous solution (v/v = 55:45, which was adjusted to pH = 3.0 with phosphoric acid.). The flow rate was set to 1.0 mL/min and the detection wavelength was 254 nm. The column temperature was retained at 30 °C.

#### 2.2.2. Method validation

The stock solution of SB at a concentration of 217.5 μg/mL was prepared by dissolving the appropriate amount of reference substance in methanol. The working standard solutions were subsequently prepared at the final concentrations of 0.0435, 0.174, 0.435, 0.87, and 2.61 μg/mL. Then, calibration curve was constructed by plotting the peak area versus the each concentration of the working standard solution. Under the above conditions, the limit of detection (S/N = 3) and the lowest quantity limit of quantitation (S/N = 10) of SB was 0.022 µg/mL and 0.0435 µg/mL, respectively.

### 2.3. Preparation and characterization of SB–PC and SB–PC–SNEDDS

#### 2.3.1. Preparation of SB–PC and SB–PC–SNEDDS

Silybin–phospholipid complex was prepared based on previous report (Song et al., 2008). Briefly, the precisely weighed SB and phospholipid with a ratio of 1:1 (w/w) were added to a round-bottom flask, and the concentration of SB was about 10 mg/mL by adding a certain amount of anhydrous ethanol, followed by stirring at 40 °C, the solution would become clear. Then, the dried residue, recovered from the solution by vacuum evaporation at 40 °C, was gathered as SB–PC. The composition of SB–PC–SNEDDS was optimized with ternary phase diagrams and consisted of 27.9% of castor oil (w/w), 10.2% of Labrasol (w/w), 14.0% of Transcutol HP (w/w), 40.9% of Cremophor RH40 (w/w), and 7.0% of SB–PC (w/w). SNEDDS was prepared by stirring the mixture at 37 °C until the solution became clear.

#### 2.3.2. Characterization of SB–PC and SB–PC–SNEDDS

The complex formation of SB–PC was evaluated by the measurement of complexation efficiency (CE). Briefly, two samples of SB–PC with approximately the same weight were dissolved in methanol and chloroform, respectively, and the content of SB in each solvent was measured by HPLC. The CE was calculated by the following formula:

$$\mathrm{CE}=\frac{W_{c}}{W_{t}}\times 100\%$$

In the above formula, *W*_c_ and *W_t_* are the amount of SB dissolved in chloroform and methanol, respectively. As both SB and SB–PC are dissolvable in methanol while only SB–PC is dissolvable in chloroform, the content of SB in methanol measured by HPLC is considered as the total content of SB (complexed and uncomplexed) in SB–PC while the content of SB in chloroform is regarded as the content of complexed SB in SB–PC.

The solubility of SB and SB–PC was measured by adding excessive amount of drug to water at 25 °C in sealed glass containers. The samples were agitated for 24 h and then centrifuged for 15 min at 8000 rpm to remove undissolved SB. Then, SB content in the supernatant was analyzed by HPLC after diluting with mobile phase. Each experiment was repeated in triplicate.

Based on Chinese pharmacopeia (2015 edition), the dissolution profiles of SB were studied by paddle method. The dissolution medium (0.1 M HCl or pH 6.8 phosphate buffer saline, 900 mL) was placed in a vessel maintained at 37 ± 1 °C and was continuously stirred at 100 rpm. At the beginning of the study, SB–PC (equivalent to 5 mg of silybin) was added in the stirring dissolution medium. At different time interval, 3 mL sample was withdrawn and centrifuged for 10 min at 8000 rpm and 3 mL fresh medium was added into the vessel immediately. The content of SB in supernatant was determined by HPLC after diluting with mobile phase.

Two hundred microliters of SNEDDS was placed in a flask and shaken gently to mix thoroughly after diluting with 50 mL of water, then the Nicomp^TM^ 380 ZLS Zeta Potential/Particle sizer was used to determine the droplet size, distribution, and *ζ*-potential. Each sample was determined in triplicate.

### 2.4. *In vitro* lipolysis study

The digesting process of SB–PC and SB–PC–SNEDDS was characterized as an *in vitro* lipolysis model with a minor modification (Khan et al., 2018). In brief, the study was carried out in a thermostatic reaction vessel set to 37 °C with an automatic burette to keep pH at 6.5 by adding NaOH solutions. During the whole experiment, the speed of magnetic stirring was set to 100 rpm.

First, the lipolysis medium used to simulate the fasted conditions in GI tract in this study was prepared as follows. As shown in Table 1, a certain amount of weighted Trizma maleate, NaCl, and CaCl_2_ was first dissolved in distilled water and adjusted to pH 6.5 by NaOH after mixing thoroughly. Then, bile salts (BSs) and PC were added and made their concentration to be 5 mM and 1 mM, respectively. Then, the above lipolysis medium was blended with different formulations and equilibrated until the lipolysis was initiated by adding the freshly prepared pancreatic lipase solution with an activity of 800USP units/mL (Khan et al., 2018).

**Table 1.** Composition of the lipolysis medium.

Substance	Initial concentration
Trizma maleate (mM)	2
Na^+^ (mM)	150
Ca^2+^ (mM)	7.5
BS (mM)	5
PC (mM)	1
Pancreatic lipase (USP units/mL)	800
SB (μg/mL)	750
Total volume (mL)	90

At each time point of 2, 5, 10, 30, 60, 90, and 120 min, 1 mL sample was taken and the lipase activity was inhibited immediately by adding 4-bromobenzeneboronic acid solution. This was followed by ultracentrifugation (Beckman XL-80, ‎Brea, CA) at 27,000×*g* for 45 min (37 °C) and the SB in the aqueous phase was determined by HPLC after dilution with mobile phase.

After that, the withdrawn samples were centrifuged and the pellets were obtained by drying at 37 °C, then the dried pellets were subjected to XRD measurement. The X-ray diffraction (D/MAXX Rigaku, Tokyo, Japan) was conducted using a graphite monochromator with Cu/Ka radiation, a voltage window of 40 kV and current density of 60 mA with a scanning rate of 4 °C/min ranging from 5 °C to 50 °C.

### 2.5. Absorption study in Caco-2 cell monolayers

#### 2.5.1. Transport across Caco-2 cells

Caco‐2 cells were grown in high-glucose DMEM containing 10% fetal bovine serum, 1% sodium pyruvate, 1% non-essential amino acids, and 1% antibiotic solution at 37 °C, 5% CO_2_, and 95% humidity. The harvested cells were seeded in 24‐well plate at 5 × 10^4^ cells/mL and cultured for 21 days. Then, cells were washed with HBSS of pH 6.0 at 37 °C for 20 min. Measured by a Millicell^®^-ER system (Millipore, Billerica, MA) before and after experiments, only cell monolayers with the transepithelial electrical resistance (TEER) value exceeding 500 Ω•cm^2^ were selected for transport study and washed with HBSS for three times before use.

For SB transport studies carried out on mature Caco-2 cell monolayers at 37 °C, test samples (0.4 mL at pH 6.0) and HBSS (1 mL at pH 6.0) were added to the donor and receiver compartment, respectively. At each time points, 200 μL samples were obtained from the receiver compartment and the same amount of HBSS (pH 6.0) were replenished immediately. The content of SB in the above obtained samples was analyzed by HPLC in triplicate.

According to the following equation, the apical to basal apparent permeability coefficient was determined by:

$$P_{\text{app}}=\frac{dC/dt\times V}{A\times C_{0}}$$

where d*C*/d*t* indicates appearance rate of SB in the basolateral side, *V*, *A*, and *C*_0_ represent the receptor well volume (mL), the membrane insert area, and the concentration of initial drug added to the donor compartment, respectively.

#### 2.5.2. Cell membrane fluidity measurement

The method was developed according to a previously described method with minor adjustments (Zhang et al., 2015). After culturing in six-well plates for at least 21 days, confluent caco-2 cell monolayers were treated with medium or medium plus different drugs which including the positive control treatments (cholesterol and benzyl alcohol). Before washed with PBS for three times, trypsinized with trypsin (1 mL/well) and pelleted for 5 min at 15 °C and 1000 g, the cells were incubated for two hours. Then, Caco-2 cells suspended in PBS (2 × 10^5^ cells/mL, 2.5 mL) were incubated and labeled with 2.5 μL of 1 mM 1,6-diphenyl-1,3,5-hexatriene (DPH) at room temperature in the dark for 30 minutes. Using a fluorescence filter lifetime and steady state spectrometer (FLS 920, EDINBURGH Instruments, Livingston, UK), fluorescence polarization was measured as excitation and emission wavelengths of 360 nm and 430 nm, respectively.

Fluorescence anisotropy (*r*) was measured according to the following equation:

$$r=\left(G_{\text{i}}I_{\text{VVi}}-I_{\text{VHi}}\right)/\left(G_{\text{i}}I_{\text{VVi}}+2I_{\text{VHi}}\right)$$

where *I*_VVi_ and *I*_VHi_ represent the fluorescence intensities recorded in the directions parallel and perpendicular to the polarized exciting light, respectively. *G* was calculated as *I*_HHi_/*I*_HVi_.

#### 2.5.3. Analysis of ZO-1 by Western blotting

The expression of tight junction protein ZO-1 effected by SB–PC and SB–PC–SNEDDS was determined by Western blotting (Zhou et al., 2017). After treated with different formulations (equivalent to 0.250 mg/mL of SB), radio-immunoprecipitation assay (RIPA) lysis buffer including protease and phosphatase inhibitors were added to homogenize cell monolayers and the supernatant was collected for protein content determination after centrifuged for 15 min at 10,000×*g* and 4 °C. Briefly, 10% SDS-polyacrylamide gels were used to separate the equal amount of total protein, which was then transferred to a nitrocellulose membrane. The membranes were blocked with Tris-buffered saline with 0.1% Tween (TBST) containing 5% dry milk for 2 h at room temperature, then these membranes were incubated with rabbit anti-ZO-1 at 4 °C overnight, followed by incubation with horseradish peroxidase (HRP)-conjugated secondary antibody at room temperature for 1 h after being washed in TBST for three times.

### 2.6. Single-pass intestinal perfusion study

The *in situ* single pass perfusion study was conducted as the methods described previously with some modifications (Singh & Pai, 2015). In order to reduce the number of rats, simultaneous perfusion in two segments (duodenum and jejunum, ileum and colon) was utilized. Briefly, 30 Sprague-Dawley (SD) rats divided into six groups were fasted overnight but had free access to water and then anesthetized with an intraperitoneal injection of pentobarbital sodium (40 mg/kg). Immediately, they were placed on a heated surface maintained at 37 ± 1 °C. When the abdominal cavity was opened, selected segments were carefully exposed, then four intestinal loops were made by cannulated at both ends including 10 cm long duodenum (1 cm distal to pyloric sphincter), 10 cm jejunum (15 cm to pyloric sphincter), 20 cm ileum (20 cm proximal to cecum), and colon (2 cm distal to cecum). The 37 °C physiological saline was used to rinse the selected segments which were attached to a peristaltic pump and covered with a piece of cotton soaked in normal saline. During the surgery, care was taken to maintain an intact blood circulation. At the start of this experiment, the isolated segments were emptied by Krebs–Ringer’s buffer at a flow rate of 0.4 mL/min for 15 min. Then, Krebs–Ringer’s buffer dissolved or dispersed with the studied formulations (SB, SB–PC and SB–PC–SNEDDS) at an SB concentration of 10 µg/mL was perfused through the isolated segments at a flow rate of 0.2 mL/min for 1 h. At the end of the experiment, the collected perfusate solution and Krebs–Ringer’s buffer adopted to rinse the segments for three times were mixed together and diluted to 25 mL with Krebs–Ringer’s buffer. Then, 1 mL of the supernatant out of 1.5 mL of above mixture centrifuged at 10,000 rpm for 10 min was diluted with equal volume of methanol and concentration of SB in the resultant solution was determined by HPLC. The length and inner of the perfused intestinal segments were also accurately measured.

Based on the following equations, the absorption rate constant (*K*_a_) and apparent permeability coefficients (*P*_app_) were calculated.

$$\begin{array}{c} K_{a}=\;\left(X_{0}-X_{t}\right)/C_{0}tV\\ P_{\text{app}}(\text{cm}/s)=Q•\;\text{ln}\;(X_{\text{in}}/X_{\text{out}})/2\pi rl \end{array}$$

In the above equations, the SB amount in the perfusate solution at 0 h and in the remaining perfusate at the end of the experiment are marked as *X*_0_ and *X_t_*, respectively. *C*_0_ is the concentration of SB at 0 h. *t*, *V*, and *Q* are represented the perfusion time, the volume of the perfused intestine segment and the flow rate, respectively. *X*_in_ and *X*_out_ are the amount of inlet and outlet drug, *r* and *l* are the radius and length of the perfused intestinal segment, respectively.

### 2.7. Intestinal lymphatic transport and systemic bioavailability of SB in anesthetized rat model

The intestinal lymphatic transport studies were conducted according to the established methods with some modifications (Chaudhary et al., 2015; Li et al., 2017). SD rats (male, 280–320 g) were fasted for 12 h with free access to water. 30 min before the experiment, each rat was fed with 1 mL of sesame oil to facilitate viewing of the mesenteric lymph duct due to its milky-white in appearance and adhesion to the mesenteric artery. At the beginning of the experiments, the rats were anesthetized by intraperitoneal injection of 200 mg kg^−1^ sodium pentobarbital, then shaved and disinfected on the right side of their necks and flanks. The jugular vein in the right neck was isolated and used to collect blood sample by cannulating with PE10 tubing. Then, the mesenteric lymph duct and duodenum in the right abdomen were exposed and cannulated with PE10 and PE50 tubing, respectively. The mesenteric lymph duct was fixed with instant cyanoacrylate adhesive. The PE50 tube inserted into the duodenum was adopted for administration and saline infusion. The body temperature of the rats was maintained by a heating pad set at 37 °C.

After surgical procedures, blank samples were collected into a heparin-rinsed Eppendorf. SB and SB–PC was dissolved and dispersed in water, the volume of which was equivalent to SNEDDS for administration, and the three formulations were administered at dosage of 80 mg/kg SB via duodenal cannula. After administration, the pre-weighed tubes were taken to gather the lymph hourly for 8 h. The blood samples were collected at 0.17, 0.33, 0.5, 1, 2, 3, 4, 6, and 8 h. Except for dosing period, the duodenum was perfused with normal saline at 2.8 mL·h^−1^ in order to maintain body hydration and intestinal lymph flow.

After centrifuging at 10,000×*g* for 10 min, the supernatant layer from the mixtures of 100 μL biological samples and 250 μL of methanol was analyzed by UPLC. Chromatography was performed using a Waters H-Class UPLC, Sample Manager-FTN, Quaternary Solvent Manager, UV Detector (Waters, Milford, MA) with a Phenomenex Luna-C_18_ column (5 μm, 4.6 mm × 150 mm) at 288 nm. The flow rate of mobile phase (water:methanol = 52:48) was 0.8 mL/min.

The cumulative lymphatic absorption of the drug over time was calculated according to the following formula:

$$X_{i}=X_{i-1}+C_{i}\times V_{i}$$

where *X_i_* was the cumulative lymphatic absorption of the drug from 0 to *i* h, *C_i_*and *V_i_* were the SB concentration and volume of lymph sample collected at *i* h, respectively.

Standard non-compartment analysis conducted with DAS2.0 was used to determine the pharmacokinetic parameters, including the plasma concentration–time curves (AUC_0–_*_t_*). The peak plasma concentration (*C*_max_) and the time of this occurrence (*T*_max_) were noted directly from the individual profiles.

### 2.8. Statistical analysis

All the data are expressed as mean ± SD. One-way analysis of variance (ANOVA) with the LSD test was applied for calculating the statistical significance between different groups and a value of *p* < .05 was considered as the minimum level of statistically significance.

## 3. Results and discussion

### 3.1. Characterization of the formulation

The CE of SB–PC was (98.2 ± 3.5) %. The SB–PC–SNEDDS gently mixed with water was used to analyze its droplet size and zeta potential, which was (92.0 ± 5.3) nm and (14.02 ± 7.12) mV, respectively.

As displayed in Table 2, SB–PC can significantly enhance SB’s water-solubility, comparing with free SB and its physical mixture with PC. As shown in Figure 1(B), the dissolution percent of SB from SB–PC was about 5- and 10-fold time higher than that from free SB in 4.5 hours in 0.1 M HCl and phosphate buffer saline (pH 6.8), respectively. The above results indicated that SB–PC could increase the dissolution of SB in both gastro and intestinal fluid.

**Table 2.** Apparent solubility of SB and SB–PC in water.

	Apparent solubility (µg/mL)
SB	41.26 ± 2.86
SB + PC	49.23 ± 3.32
SB–PC	82.14 ± 4.55

### 3.2. Percentage of lipolysis

**Figure 2.**
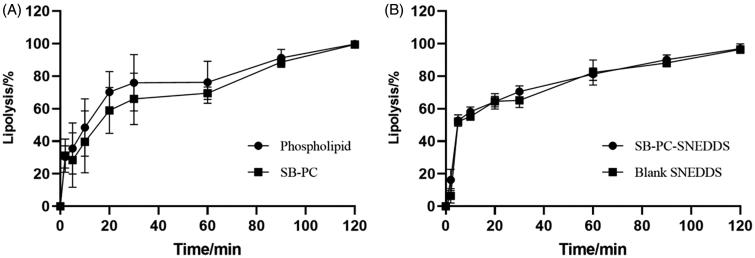
Time dependence of the lipase-mediated digestion of SB–PC and SNEDDS (*n* = 3).

The extent of lipolysis was monitored by recording the volume of consumed NaOH solutions and it was assumed that the lipolysis percentage was 100% at 2 h. Figure 2 shows that the lipolysis percentage over time of SB–PC and SB–PC–SNEDDS was similar with that of phospholipid and blank SNEDDS, respectively. It could be concluded that the property of the used lipid in the formulation was the main influencing factor of the lipolysis of PC and SNEDDS.

### 3.3. Analysis of isolated digestion phases *in vitro* lipolysis

#### 3.3.1. Analysis of drugs in the aqueous phase

**Figure 3.**
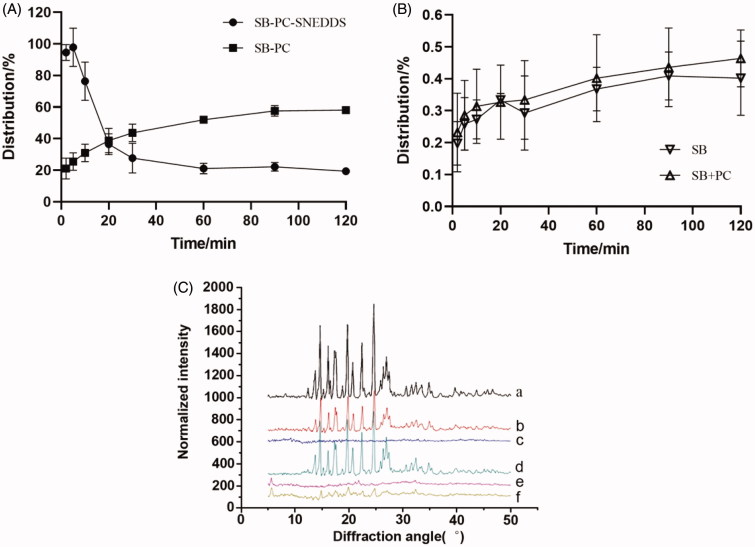
Analysis of isolated digestion phases *in vitro* lipolysis. (A) Distribution (%) of SB in the liquid phase throughout the *in vitro* lipolysis of the SB–PC and SB–PC–SNEDDS. (B) Distribution (%) of SB in the liquid phase throughout the *in vitro* lipolysis of the SB and SB + PC. (C) XRD patterns of (C-a) SB-starting crystalline SB used in SNEDDS; (C-b): SB (lipolysis)-pellet from the lipolysis of SB; (C-c) SB–PC (lipolysis)-pellet from the lipolysis of SB–PC; (C-d) SB + PC (lipolysis)-pellet from the lipolysis of the physical mixture of SB and PC; (C-e) SB–PC–SNEDDS (lipolysis)-pellet from the lipolysis of SB-SNEDDS; (C-f) blank SNEDDS (lipolysis)+SB-pellet from the lipolysis of blank SNEDDS spiked with SB.

The distribution of drugs during lipolysis is presented in Figure 3(A,B) and Table 3. The distribution of SB–PC in the aqueous phase gradually increased to 59% over time in 120 min. In lipolysis process, phospholipids were first hydrolyzed to free fatty acids and 2-acyl and 1-acyl lysophospholipids, and glycerophosphate acid was the usual further hydrolysis product of both lysophospholipids (Zuidam & Crommelin, 1995); these hydrolysates of phospholipids could not reduce the solubilization capacity of SB in the media.

**Table 3.** Comparison of concentration of SB in the liquid phase of *in vitro* lipolysis of the SB, SB–PCx, and SB–PC–SNEDDS (*n* = 3).

	*C*_2h_ (μg/mL)	*C*_max_ (μg/mL)
SB	2.98 ± 1.12	2.92 ± 1.12
SB–PC	442.94 ± 16.31	442.94 ± 16.31
SB + PC	3.64 ± 0.59	3.64 ± 0.59
SB–PC–SNEDDS	143.83 ± 15.17	736.21 ± 91.61

For the lipolysis process of SNEDDS, because of the fine dispersion of SNEDDS, the distribution of SB in the aqueous phase increased to 100% in the first 5 min. In this work, Ca^2+^-ions were added as a bolus at the start of lipolysis. As prompted by Alaadin’s work (Alayoubi et al., 2018), the hydrolysis of triacylglycerides from SNEDDS might be completed quickly and the structure of most SNEDDS would be destroyed in short time, so precipitation occurred and the distribution of SB in aqueous phase rapidly decreased to about 30% in the next 15 min. The precipitation was calcium soaps of free fatty acids reacted with Ca^2+^-ions during *in vitro* lipolysis. However, the distribution of SB from SB–PC–SNEDDS in the aqueous phase was lower than that of SB–PC in 20 min later, attributing to the SNEDDS digestion products which might form structure with a lower solubilizing effect. The complex of fatty acids with calcium also could further reduce the solubilizing capacity (Sassene et al., 2010).

The SB concentrations in aqueous dispersed phase for SB–PC and SB–PC–SNEDDS were 148 times and 247 times (*C*_max_) higher respectively than that for SB which was similar to the physical mixture (SB and PC). These results reflected that the dissolution of SB was improved by the formation of not only SB–PC but also SB–PC–SNEDDS.

#### 3.3.2. Analysis of drugs in the pellet phase

Precipitation collected after lipolysis was characterized by XRD analysis (Figure 3(C)). The free SB drug pattern exhibited several characteristic reflections of the crystalline SB. XRD patterns of the precipitation obtained after lipolysis for SB, the mixture of SB and PC, and the lipolysis of blank SNEDDS spiked with SB were similar with crystalline SB. However, both SB–PC and SB–PC–SNEDDS showed no presence of characteristic crystalline peaks of SB in the pellet, suggesting the presence of a high energy amorphous state with better dissolution and absorption.

### 3.4. Absorption study by Caco-2 cells monolayers

#### 3.4.1. Transport across Caco-2 cells

**Figure 4.**
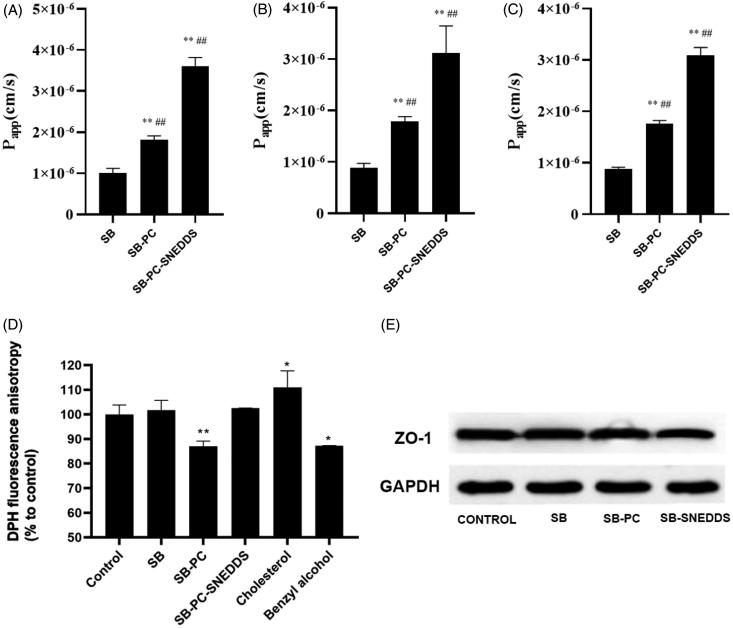
Absorption study by Caco-2 cells monolayers. (A–C) Transport (*P*_app_) of SB, SB–PC, and SB–PC–SNEDDS in Caco-2 cells (A: 10 μg/mL; B: 20 μg/mL; C: 30 μg/mL). **p < .01, compared with SB; ^##^*p* < .01, compared with SB–PC. (D) The membrane fluidity (inverse of fluorescence anisotropy) of Caco-2 cell membranes for SB, SB–PC, and SB–PC–SNEDDS (*n* = 4, **p < .01). (E) Western blotting analysis of ZO-1 proteins of Caco-2 cell treated with blank solvent, SB, SB–PC, and SB–PC–SNEDDS.

As shown in Figure 4(A–C), the SB transport across Caco-2 cells at 37 °C could been significantly increased by SB–PC and SB–PC–SNEDDS by comparison with free SB at concentration of 10, 20, and 30 μg/mL. *P*_app_ was also noticeably enhanced to about 2-fold and 3.5-fold (*p* < .01), indicating that PC and SNEDDS could facilitate the transport of drug significantly.

The results of the transport from apical to basolateral (AP-BL) and from basolateral to apical (BL-AP) are displayed in Table 4, which showed that the ratios of *P*_app BL-AP_/*P*_app AP-BL_ of free SB, SB–PC, and SB–PC–SNEDDS were 17.43, 9.50, and 6.37, respectively. This result indicated that SB–PC and SB–PC–SNEDDS could noticeably inhibit P-gp efflux. As shown in Table 5, cyclosporin A (CsA) could significantly improve the transport of AP-BL and reduce the transport of BL-AP (*p* < .01) while these effects could be reduced by SB–PC and SB–PC–SNEDDS, suggesting that SB was a substrate of P-gp as revealed by previous studies (Ferreira et al., 2019) and further confirming that SB–PC and SB–PC–SNEDDS could inhibit P-gp efflux. It is likely due to the change of transmembrane way for SB–PC and some surfactants in the SNEDDS which could inhibit P-gp efflux.

**Table 4.** Effect of direction on the *P*_app_ of SB by Caco-2 cells (*n* = 3).

Drugs	*P*_app_ × 10^–6^ (cm/s)		Ratio *P*_app BL-AP_/ *P*_app AP-BL_
	Apical–basolateral	Basolateral–apical	
SB	1.02 ± 0.08	17.80 ± 0.76**	17.43
SB–PC	1.83 ± 0.06	17.36 ± 0.55**	9.50
SB–PC–SNEDDS	3.60 ± 0.21	22.94 ± 1.31**	6.37

** *p* < .01 indicates a statistically significant difference when compared with AP-BL.

**Table 5.** Effect of cyclosporin A (CsA) on the *P*_app_ of SB by Caco-2 cells (*n* = 3).

Direction	Drugs	*P*_app_ × 10^–6^ (cm/s)		Ratio CsA/control
		CsA (10 μg⋅mL^–1^)	Control	
A–B	SB	3.56 ± 0.11**	1.02 ± 0.08	3.49
	SB–PC	3.65 ± 0.27**	1.83 ± 0.06	2.00
	SB–PC–SNEDDS	4.48 ± 0.83**	3.60 ± 0.21	1.24
B–A	SB	7.84 ± 0.66**	17.80 ± 0.76	0.44
	SB–PC	9.38 ± 0.55**	17.36 ± 0.55	0.54
	SB–PC–SNEDDS	16.22 ± 0.89**	22.94 ± 1.31	0.71

** *p* < .01 indicates a statistically significant difference when compared with control.

#### 3.4.2. Cell fluidity measurements

The effects of free SB, SB–PC and SB–PC–SNEDDS on the fluidity of the Caco-2 cell membrane were assessed by detecting the DPH fluorescence anisotropy. The decrease in DPH fluorescence anisotropy implies the increase of membrane fluidity. In this study, cholesterol and benzyl alcohol were chosen as positive controls, which would decrease and increase the membrane fluidity of Caco-2 cells, respectively. As shown in Figure 4(D), the DPH fluorescence anisotropy of Caco-2 cells increased and decreased by 11.0% and 12.8%, which were exposed to 25 µM cholesterol and 30 mM benzyl alcohol for 2 h, respectively. SB–PC significantly decreased the DPH fluorescence anisotropy, implying the increase membrane fluidity at the hydrophobic core of the bilayer which was easier to across for lipophilic solutes (Souza et al., 2003). The difference of the DPH fluorescence anisotropy between SB and SB–PC–SNEDDS was not significant.

#### 3.4.3. Analysis of ZO-1 by Western blotting

After treatment with SB, SB–PC, or SB–PC–SNEDDS for 2 h, the amount of ZO-1 in Caco-2 monolayers was detected by Western blotting in this paper. As revealed by Figure 4(E), SB–PC–SNEDDS group can lower the amount of ZO-1 by comparison with control groups, while there was no significant difference of ZO-1 expression among SB, SB–PC, and control groups, which indicate the tight junction integrity of Caco-2 cell monolayers might been opened by SB–PC–SNEDDS due to some surfactants used in present SNEDDS such as Labrasol which can redistribute of ZO-1 as reported (Wu et al., 2015).

### 3.5. Single-pass intestinal perfusion studies

**Figure 5.**
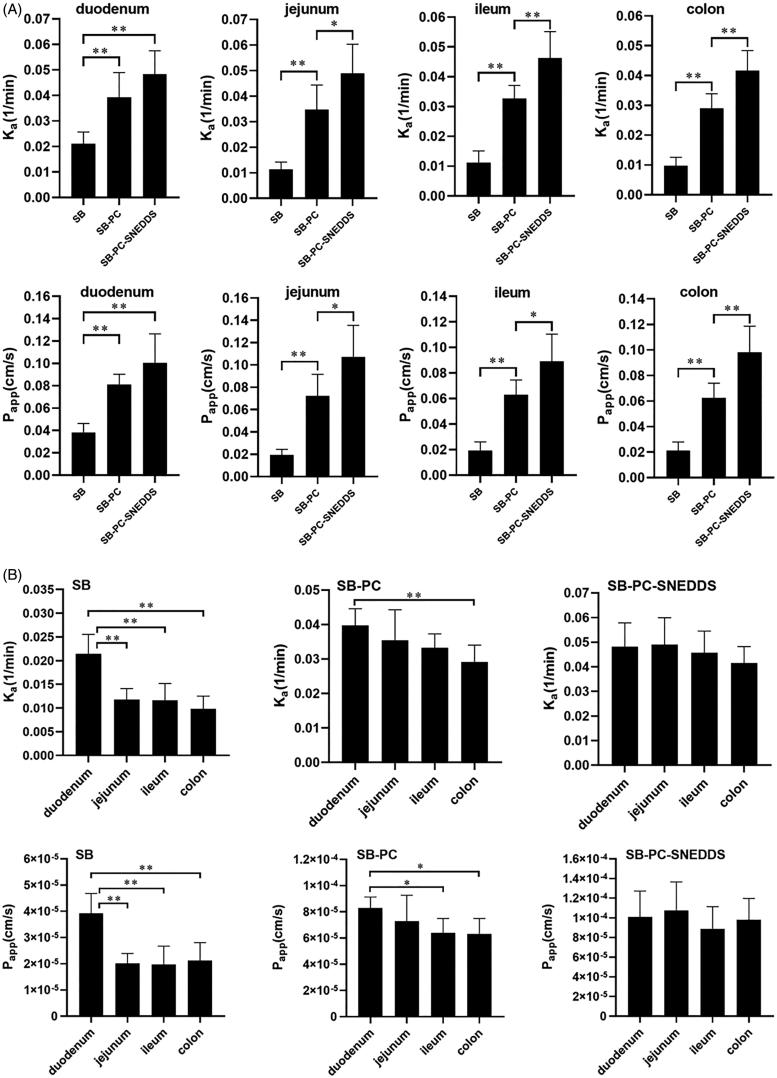
The absorption rate constant (*K*_a_) and the apparent permeability coefficient (*P*_app_) for GTP obtained by *in situ* perfusion in SPIP model. (A) Comparison among three drugs. (B) Comparison among four different intestinal segments. Data presented as mean ± SD (*n* = 5). *p  <  .05, **p < .01.

According to results of *in situ* perfusion in SPIP model, the values of *K*_a_ and *P*_app_ from these three drugs are presented in Figure 5(A). Compared to free SB, *K*_a_ and *P*_app_ values of SB–PC and SB–PC–SNEDDS were significantly higher in all the intestinal segments (*p* < .01), and SB–PC–SNEDDS exhibited significantly higher values of *K*_a_ and *P*_app_ than SB–PC in four intestinal segments except for duodenum (*p* < .05 or *p* < .01). In addition, the comparison of *K*_a_ and *P*_app_ values among different intestinal segments were also carried out and described in Figure 5(B). The absorption of SB (*K*_a_ and *P*_app_) was duodenum > jejunum ≈ ileum ≈ colon (*p* < .01), and for SB–PC was duodenum > colon (*p* < .05). However, the differences of both *K*_a_ and *P*_app_ values for SB–PC–SNEDDS among four segments were not significant.

These results implied that the intestinal absorption of SB–PC–SNEDDS and SB–PC was significantly better than that of SB. While in ileum and colon, SB–PC–SNEDDS showed higher absorption than SB–PC. The absorption of SB–PC–SNEDDS and SB–PC was colon > ileum>(jejunum and duodenum). The best absorption occurred when SB was perfused in duodenum (*K*_a_ 2.16 × 10^−2^/min; *P*_app_ 3.97 × 10^−5^ cm/s), indicating that duodenum was the main absorption sites for SB. The difference between duodenum and other segments decreased. For SB–PC–SNEDDS, however, the good absorption showed in all the four intestinal segments, which might contribute to the improved absorption.

### 3.6. Intestinal lymphatic transport and systemic bioavailability of SB in the anesthetized rat model

**Figure 6.**
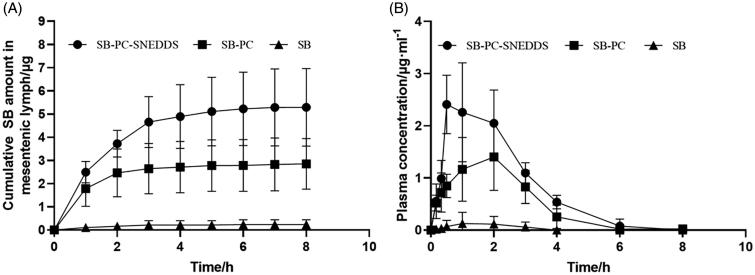
Intestinal lymphatic transport and systemic bioavailability of SB in the anesthetized rat model. (A) The cumulative transport of SB in mesenteric lymph as a function time following duodenal administration to rats (*n* = 3). (B) Mean plasma concentration versus time profiles for SB following duodenal administration to rats.

In the present work, the contents of SB by lymphatic transport for the three formulations were analyzed and expressed as the cumulative SB amount versus time, which is shown in Figure 6(A). After intraduodenal administration of SB, SB–PC, and SB–PC–SNEDDS for 8 h, the total SB amount in the mesenteric lymph was (0.23 ± 0.21) μg, (2.85 ± 1.09) μg, and (5.29 ± 1.67) μg, respectively, which indicate greatly enhanced lymphatic absorption by SB–PC and SB–PC–SNEDDS (*p* < .05). After intraduodenal administration of SB, SB–PC, and SB–PC–SNEDDS, the function of portally absorbed SB concentration over time is described in Figure 6(B), and the pharmacokinetic parameters are shown in Table 6. The values of *C*_max_ and AUC_0–∞_ were SB < SB–PC < SB–PC–SNEDDS (*p* < .05).

**Table 6.** Comparison of intestinal lymphatic transport and bioavailability of SB in lymph-cannulated rats after intraduodenal administration in SB, SB–PC, and SB–PC–SNEDDS.

Parameter	SB	SB–PC	SB–PC–SNEDDS
*T*_max_/h	1.500 ± 0.707	1.333 ± 0.577	1.167 ± 0.764
*C*_max_/μg·mL^–1^	0.219 ± 0.221	1.533 ± 0.576*	3.027 ± 0.323**^,##^
AUC_0–∞_/μg·mL^–1^·h	0.398 ± 0.689	5.034 ± 1.388**	7.168 ± 0.808**^,#^
AUC_0–8 h_/μg·mL^–1^·h	0.247 ± 0.428	3.816 ± 1.745**	6.553 ± 1.306**^,#^
Fr (%)		1265.9	1802.5

*p < .05 and **p < .01 indicate a statistically significant difference when compared with SB.

^#^*p* < .05 and ^##^*p* < .01 indicate a statistically significant difference when compared with SB–PC.

The lymphatic transport route was an important way for lipid-based formulations, which was increasingly emphasized in recent years. It can greatly improve bioavailability of drugs by avoiding the hepatic first-pass metabolism (Managuli et al., 2018; Vishwakarma et al., 2019). In the lymph duct-cannulated rats, the relative bioavailability of SB–PC and SB–PC–SNEDDS was 1265.9% and 1802.5%, respectively. SB–PC displayed impressive enhancement of the water-solubility and dissolution and improvement of the biological effect of SB. Phospholipid was an important component of cell membrane, having the effects of keeping cell membrane fluidity. Phospholipid complex technology produces a little cell, whereby SB was protected from destruction by gastric secretions and gut bacteria (Bombardelli et al., 1991; Kumar et al., 2010). For SNEDDS, the surfactants could greatly enhance the oral absorption by dispersing the lipid formulation in the GI tract and then forming droplets with nanosize (Lu et al., 2018; Kanwal et al., 2019). The cosurfactants could help to improve the solubility of the hydrophilic surfactant or the drug in the lipid base.

## 4. Conclusions

The PC and its SNEDDS significantly enhance the hydrophilicity and dissolution of SB, and the precipitated drug in a high energy amorphous form during digesting might have better GI tract fluid solubility, dissolution, and absorption. For PC, it can improve the oral bioavailability by promoting the intestinal absorption, inhibiting the P-gp efflux and increasing the membrane fluidity and lymphatic absorption. For PC–SNEDDS, the possible improvement mechanisms of oral bioavailability were mainly due to the inhibiting P-gp efflux, opening cell tight junctions and enhancing lymphatic absorption. This study provides a meaningful way for research and development of water insoluble drugs by improving their oral bioavailability through the combination of PC and SNEDDS.
